# “Noninfective Endocarditis”: A Case Report of Hereditary Coagulation Disorders in a 28-Year-Old Male

**DOI:** 10.3390/diagnostics10060384

**Published:** 2020-06-08

**Authors:** Gregory Reid, Luca Koechlin, Oliver Reuthebuch, Florian Rüter, Helmut Hopfer, Friedrich Eckstein, David Santer

**Affiliations:** 1 Department of Cardiac Surgery, University Hospital Basel, 4031 Basel, Switzerland; gregory.reid@usb.ch (G.R.); luca.koechlin@usb.ch (L.K.); oliver.reuthebuch@usb.ch (O.R.); florian.rueter@usb.ch (F.R.); friedrich.eckstein@usb.ch (F.E.); 2 Institute of Medical Genetics and Pathology, University Hospital Basel, 4031 Basel, Switzerland; helmut.hopfer@usb.ch

**Keywords:** acute coronary syndrome, anticoagulation, genetic disorders, thrombosis, ultrasound, valve replacement

## Abstract

We report a case of a young male who presented with acute limb ischemia after sport. With no prior history of disease, a non-infective endocarditis of the native aortic valve was diagnosed. After surgical valve replacement, the patient suffered from acute myocardial ischemia under phenprocoumon therapy. Anti-coagulant monitoring was subsequently changed to Factor II analysis after a rare Factor VII deficiency and prothrombin mutation (G20210A) was diagnosed.

## 1. Introduction

The foremost working diagnosis, when a patient presents with acute limb ischemia is an embolus of cardiac source. A large study of 822 cases in 2012 found 79.9% to be of cardiac source, the absolute majority comprising of heart valve disease and atrial fibrillation [1]. The symptoms are often clear, and the 5 P’s (pain, pallor, paresthesia, pulselessness, paralysis) are learned early on by medical students. The diagnostic algorithm includes investigating other potential sources such as atherosclerosis, or emboli originating from an aneurysm sack. Differential diagnosis include trauma, thrombosis following intervention, as well as low flow state and a history of peripheral artery disease. Emboli originating from a heart valve are most commonly due to endocarditis, followed by malignancy. A nonbacterial thrombotic endocarditis (NBTE) is a sub form of culture-negative endocarditis, associated with a number of conditions such as advanced malignancy [2], autoimmune diseases, like systemic lupus anticoagulants, and rheumatoid arthritis. Inherited hypercoagulable conditions include Factor V Leiden, prothrombin gene mutation or an imbalance of clotting and anti-clotting factors.

## 2. Case Presentation

**Figure 1:**
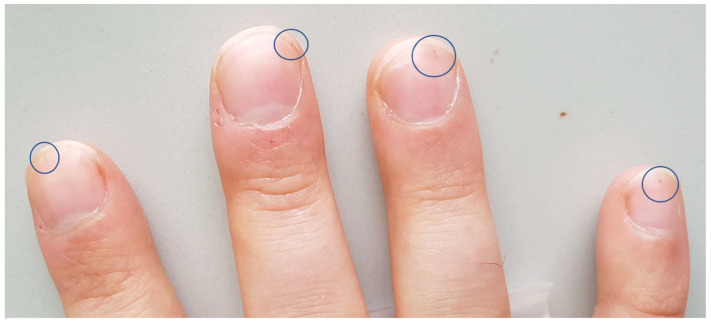
Clinical symptoms. Splinter hemorrhages discretely visible on the nails of the right hand (circles) were one of the first symptoms presented by the patient.

A 28-year-old male experienced fever and increasing unilateral calf pain after a football game and presented himself to the emergency department the following day. The physical examination showed typical signs of a peripheral arterial occlusion, as well as splinter hemorrhages of the fingernails (Figure 1). The rest of the physical examination was inconspicuous and there were no signs of infection in the otherwise normal blood tests. The patient had no relevant personal medical history, nor family medical history. He had no history of drug or alcohol consumption. The patient’s written consent for use of his data and tissue for research purposes and the subsequent publication were obtained.

Ultrasound scan diagnosed an acute embolic closure of the left popliteal artery and the patient underwent immediate embolectomy. Histological examination of the embolus showed thrombotic material without any sign of microorganisms. Further diagnostic workup during hospitalization displayed a visible vegetation with a cross diameter of 6 mm on the bicuspid aortic valve in transthoracic echocardiography and was subsequently confirmed in transesophageal echocardiography (TEE) along with a small patent foramen ovale (PFO). A TEE was seen as a supplemental test for evaluation for cardiovascular source of embolus with no identified noncardiac source [3]. Phenprocoumon was started in therapeutic dose. All blood cultures came up negative and the patient had no neurological symptoms. Empiric antibiotic treatment was initiated.

**Figure 2:**
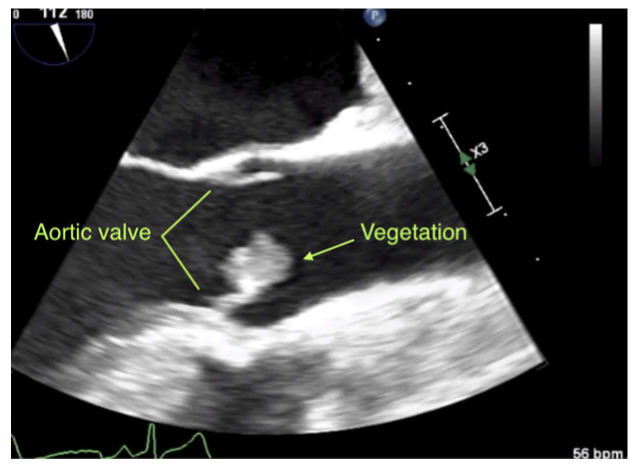
Ultrasound video (still). Transesophageal echocardiography performed in the long-axis view at midesophageal level. A visible echogenic mass, consistent with a vegetation of 12 × 12 × 10 mm^3^ adhered to the native aortic valve.

Six weeks later, TEE demonstrated a sudden progressive growth of the vegetation to 12 × 12 × 10 mm^3^ and new moderate aortic insufficiency (Figure 2; Supplementary Video S1). Due to the lack of regression, a more complex bleeding disorder seemed unlikely.

**Figure 3:**
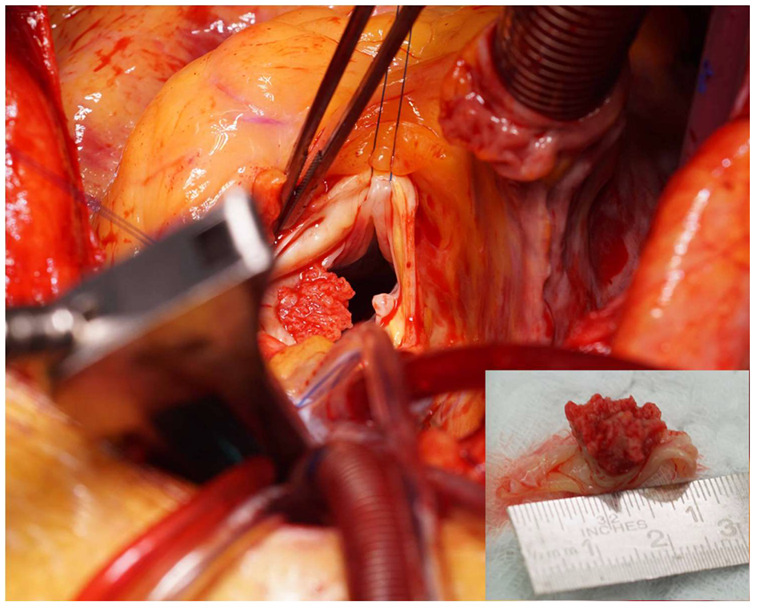
Intraoperative view. Intraoperative view showing a large vegetation on the aortic valve and close-up of the excised leaflet (inset). Two leaflets fused to form a functionally bicuspid valve.

**Figure 4:**
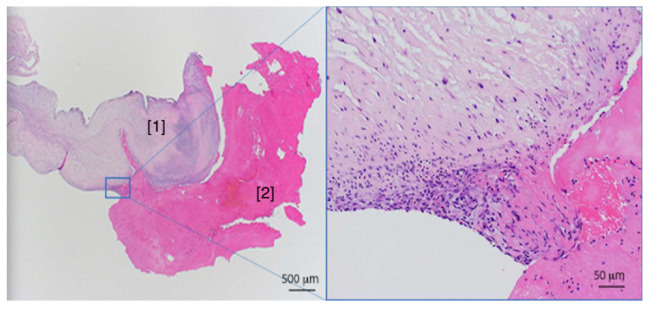
Histological examination. Histological examination of the excised aortic valve (hematoxylin and eosin stain) shows a florid inflammation consisting mainly of neutrophilic leucocytes. [1] Leaflet and [2] attached fibrinous material.

The indication for urgent aortic valve reconstruction was given by the interdisciplinary heart team. Intraoperatively, the valve was tricuspid with a large vegetation fusing and destructing two leaflets, creating a functionally bicuspid valve (Figure 3). Therefore, a mechanical aortic valve (Medtronic Open Pivot™ AP 360^®^, 28 mm) was implanted. The histopathological analysis of the vegetation, also using PCR analysis, showed no identification of common or rare pathogens, or organisms. Light microscopy revealed a destructive, ulceropolyposis of the native valve combined with a florid inflammation composed predominantly of leucocytes and fibrin and again no signs of bacterial infection (Figure 4). The patient recovered well and was discharged after a short hospitalization under phenprocoumon therapy with target international normalized ratio (INR) values of 2.0–3.0. Subsequent genetic testing as an outpatient revealed a hereditary heterozygous prothrombin-mutation (G20210A-Mutation).

Seven months later, the patient presented himself again to the emergency ward with chest pain after cycling. High-sensitivity Troponin T was increased to a maximum value of 1521 ng/L (0–14 ng/L) and creatine kinase myocardial band (CK-MB) to 76 µg/L (0–5 µg/L). The coronary angiogram showed multiple, distal coronary embolisms with no signs of atherosclerosis (Supplementary Video S2). In echocardiography a well-functioning mechanical valve prosthesis without any signs of adhering material was observed and there was no change in the left ventricular ejection fraction (LVEF) (60%). No intervention was performed, and the patient was monitored in the intensive care unit. Repeat blood tests including a full coagulation factor panel showed a Factor VII deficiency leading to false high INR values. Anticoagulation monitoring was subsequently changed from INR monitoring to Factor II analysis with target values of 20–25%.

The patient was discharged after a short hospitalization and at the time of writing (May 2020), had fully recuperated at the 18-month cardiological follow-up. Factor VII deficiency is an indication for family screening [4], however the patient and his family were unable to attend hematologic follow-ups.

## 3. Discussion

Prothrombin (Factor II) is a vitamin K-dependent protein synthesized in the liver and in the coagulation cascade; it is cleaved to form the terminal enzyme thrombin (Factor IIa), that in turn proteolytically cleaves fibrinogen to fibrin. A G20210A mutation results from a base substitution in a non-coding region of the prothrombin gene leading to roughly 30% higher plasma prothrombin levels in homozygote carriers [5]. The overall prevalence is estimated around 2% in Caucasian populations [6] and it is often seen in combination with Factor V Leiden (FVL). Case-control studies have demonstrated that affected individuals have an increased risk of venous thromboembolism (VTE; odds ratio of 3.1) and even further increase if both mutations are present [7]. Meta-analyses have demonstrated the minor significance of the mutation as a risk factor for arterial thrombosis, raising the relative risk to 1.31 [8]. Mutation could be suspected in individuals with a VTE at an early age or unusual site, or a familial thrombophilia. Routine testing is not recommended [9]; however, in these patients, an overlap between elevated plasma prothrombin levels, INR as well as activated partial thromboplastin time (aPTT) and the upper range of reference values is commonly observed. Therefore, normal results might cover pathologic values in these patients. In patients presenting at an unusual age, obtaining a full blood sample before commencing anticoagulation should be considered. Data on this subject are scarce, but patients with suspicious clinical findings, relevant family history or lack of etiology of the embolism might benefit from hematological testing. The presence of a G20210A mutation per se is not an indication for anticoagulation after a thromboembolic event, however, it should be taken into consideration in aggravating circumstances, such as pregnancy, surgery or acute medical illness [10]. In cases necessitating permanent oral anticoagulation, such as a mechanical mitral valve replacement, the recommendations do not differ. Blood coagulation monitoring of the outpatient should include Factor II analyses, alongside INR, as this directly represents the plasma levels of prothrombin. A recent case report by Arletti et al. [11] proposes treatment with direct oral anticoagulants (DOACs) for patients suffering from atrial fibrillation and moderate Factor VII deficiency. They highlight again the benefit in an outpatient setting, as DOACs have a differing mechanism of action than phenprocoumon, raising the question if this could be a non-conform treatment option [12] for the presented patient, despite the implanted mechanical valve. Retrospective analysis of the pre-operative coagulation panel showed an off-balance in the Factor II (77%) and Factor VII (38%) levels. This discreet sign could have led to further investigation of possible hereditary deficiencies.

## 4. Conclusions

Thrombotic vegetations can clinically present as endocarditis. Treatment with full-dose IV unfractionated heparin or subcutaneous low-molecular-weight heparin is suggested (evidence class II-C) [13] and early surgery is reasonable in patients with persistent vegetations despite antibiotic therapy (evidence class IIa-B) or who have mobile vegetations greater than 10 mm in length (evidence class IIb-B) [14]. Prothrombin mutation (G20210A) has a relatively high prevalence and is often seen in conjunction with other coagulation disorders such as Factor V Leiden and Factor VII deficiency. Factor II analysis alongside INR and aPTT monitoring for oral anticoagulant therapy monitoring in these patients should be considered and implemented from the beginning.

## Supplementary Materials

Supplementary materials can be found at https://www.mdpi.com/2075-4418/10/6/384/s1, Video S1: Ultrasound video. Transesophageal echocardiography performed in the long-axis view at midesophageal level. A visible echogenic mass, consistent with a vegetation of 12 × 12 × 10 mm^3^ adheres to the native aortic valve. Video S2: Coronary angiogram of the left coronary artery. A diagnostic coronary angiogram depicts a lesion in the apical section of the left anterior descending artery (LAD) and no visible sclerosis. No stenting was performed.
